# Pembrolizumab‐Induced Myositis: Diagnostic and Therapeutic Challenges From Two Case Reports and a Narrative Review

**DOI:** 10.1002/cnr2.70534

**Published:** 2026-06-24

**Authors:** Francesca Rifaldi, Niccolò Leandro Alessio, Francesca Caspani, Elena Bolzacchini, Monica Giordano, Paolo Pedrazzoli

**Affiliations:** ^1^ Department of Internal Medicine and Medical Therapy University of Pavia Pavia Italy; ^2^ Department of Oncology, Comprehensive Cancer Center IRCCS Policlinico San Matteo Foundation Pavia Italy; ^3^ Department of Oncology Sant' Anna Hospita, ASST Lariana San Fermo della Battaglia Italy; ^4^ Oncology Unit ASST Sette Laghi Varese Italy

**Keywords:** endometrial cancer, immune‐related toxicity, lung cancer, myositis, pembrolizumab

## Abstract

**Background:**

Pembrolizumab, an anti‐PD‐1 immune checkpoint inhibitor, has transformed the treatment of multiple solid tumors. However, it can rarely cause immune‐related myositis, which may involve limb, bulbar, and respiratory muscles, posing a risk of severe morbidity and mortality. Early recognition and management are essential. We report two cases of pembrolizumab‐induced myositis diagnosed and treated at our institution. A literature search of MEDLINE/PubMed was conducted to identify English‐language case reports, focusing on clinical presentation, diagnostics, management, and outcomes.

**Cases:**

The first case involved a 74‐year‐old woman with advanced lung adenocarcinoma who developed grade 4 myositis affecting limb and phonatory muscles; intravenous methylprednisolone combined with IVIG led to full recovery. The second case involved a 78‐year‐old woman with advanced endometrial carcinoma who developed grade 3 limb‐girdle myositis, successfully treated with oral prednisone. Literature review (11 case reports) indicated that most patients were older males with lung cancer, presenting with proximal muscle weakness and elevated CK. Bulbar or ocular involvement was linked to a higher risk of myocarditis and respiratory complications. Early corticosteroid therapy, with IVIG for severe cases, was consistently effective.

**Conclusions:**

Pembrolizumab‐induced myositis is a rare but potentially life‐threatening adverse event. Recognition of characteristic clinical features, prompt immunosuppressive therapy, and multidisciplinary care are critical for favorable outcomes. Additional studies are needed to establish standardized diagnostic criteria and optimal treatment protocols.

## 1 Introduction

Immune checkpoint inhibitors (ICIs) have revolutionized cancer therapy by enhancing anti‐tumor immune responses. Pembrolizumab, a humanized anti‐PD‐1 monoclonal antibody, is widely used as monotherapy or in combination regimens across multiple solid tumors, demonstrating improved progression‐free and overall survival [1].

Despite its efficacy, pembrolizumab can rarely trigger immune‐related adverse events (irAEs). Among these, myositis—an autoimmune inflammatory condition of skeletal muscles—is exceptionally uncommon but can be life‐threatening when it involves bulbar or respiratory muscles or is associated with myocarditis [2, 3]. Prompt recognition and treatment are critical to prevent severe morbidity or death.

This study aims to report two cases of pembrolizumab‐induced myositis managed at our institution, Sant'Anna Hospital (San Fermo della Battaglia, Como, Italy) highlighting their clinical presentation, diagnostic work‐up, and therapeutic strategies, and to perform a narrative review of pembrolizumab‐induced myositis in the published literature, summarizing clinical features, management approaches, and outcomes. By combining institutional experience with published evidence, this report seeks to improve awareness and inform clinical management of this rare but potentially severe irAE.

## 2 Methods

This study is a narrative review of pembrolizumab‐induced myositis. Two patients diagnosed at our institution were included, both evaluated with comprehensive clinical, laboratory, and imaging assessments. Management strategies were guided by disease severity, CTCAE grading, and multidisciplinary consultation with neurologists, rheumatologists, and oncologists.

A literature search of the MEDLINE/PubMed database was performed for the period 2020–2025 using the keywords: “pembrolizumab‐induced myositis,” “pembrolizumab and myositis,” “immune checkpoint inhibitor myositis,” and related terms. Only English‐language publications were considered. References from selected articles were manually screened to identify additional relevant reports.

Inclusion criteria for the narrative review were case reports or series reporting myositis directly attributed to pembrolizumab, including clinical and laboratory description (e.g., CK elevation, EMG findings, imaging, or biopsy) and documented therapeutic approach and outcomes. From the search, 39 articles were identified, of which 11 were pembrolizumab‐specific case reports. Nine cases were summarized in Table 1 for direct comparison with our two institutional cases.

**TABLE 1:** Clinical and demographic characteristics of reported cases of immune checkpoint inhibitor (ICI)‐associated myopathies in patients with solid tumors.

Author/year	Age/sex	Tumor type	CK (U/L)	ICI regimen	Cycles to onset	Clinical features	Autoantibodies	irAEs	Treatment	Outcome
Claus et al. 2019 [4]	57/M	Advanced lung adenocarcinoma	11 796	Pembrolizumab	1	Necrotizing myositis (legs)	NA	—	Corticosteroids	Partial recovery
Yamano et al. 2024 [5]	59/F	Advanced lung adenocarcinoma	NA	Pembrolizumab + Chemotherapy	1	Myositis + cholangitis	NA	Immune cholangitis	Corticosteroids + MMF/Azathioprine	Deceased
Soman et al. 2022 [6]	70/F	Advanced lung adenocarcinoma	3663	Pembrolizumab	1	Myositis + myocarditis + myasthenia	NA	Myocarditis, MG	Corticosteroids	Deceased
Takahashi et al. 2023 [7]	70/F	Advanced endometrial cancer	915	Pembrolizumab	1	Right ptosis and quadriceps muscle weakness	Anti‐striated	Ocular involvement	Plasma exchange + oral corticosteroids	Complete recovery
Haddox et al. 2017 [8]	78/M	Metastatic melanoma	1284	Pembrolizumab	2	Bulbar weakness, ptosis, dysphagia, respiratory failure, proximal muscle weakness	Striational antibodies	Myocarditis	Prednisone + plasma exchange	Deceased
Kobak 2019 [9]	73/M	Advanced lung adenocarcinoma	NA	Pembrolizumab	2	Fasciitis and polymyositis	NA	Arthritis, rash	Prednisolon 16 mg/day	Full recovery
Peverelli et al. 2020 [10]	60/M	Advanced lung squamous cell carcinoma	20	Pembrolizumab	NA	Myositis of eyes neck and legs	—	Myocarditis	Corticosteroids	Deceased
Kamo et al. [11]	72/F	Advanced lung adenocarcinoma	1817	Pembrolizumab	2	Systemic myositis (ocular and cervical)	—	MG‐like	Corticosteroids	Partial recovery

*Note:* This table summarizes, for each case: Author/year of publication, patient age and sex, tumor type and stage, creatine kinase (CK) levels (U/L), ICI regimen and number of cycles before myopathy onset, clinical features of myopathy/muscle involvement, associated autoantibodies when available, concurrent immune‐related adverse events (irAEs), specific treatments administered, and final clinical outcome. The data provide an overview of severe myopathies, including cases with concomitant myasthenia gravis or extra‐muscular involvement, highlighting clinical presentations, management strategies, and patient outcomes.

## 3 Case Report 1

A 74‐year‐old woman with a history of smoking, hypertension, vasculopathy, and coronary artery disease was diagnosed with lung adenocarcinoma. She had completed five cycles of first‐line pembrolizumab, achieving a partial response in both the primary lung lesions and thoracic lymph nodes. Her baseline performance status was ECOG 1.

In December 2024, she was admitted to the Department of Oncology at Sant'Anna Hospital, San Fermo della Battaglia (Italy) for pneumonia and treated with piperacillin/tazobactam and supplemental oxygen, leading to complete resolution after 8 days.

Approximately 2 months after her last pembrolizumab infusion, she developed burning pain and progressive weakness in the lower limbs. Over the following days, weakness extended to the upper limbs, accompanied by dysphonia. Brain CT scan ruled out metastatic lesions.

Laboratory evaluation revealed markedly elevated CK (4359 IU/L), ESR 76 mm/h, weakly positive ANA (AC8‐AC10), positive PL‐7 antibodies, and borderline ENA antibodies. Electromyography (EMG) demonstrated a myopathic pattern affecting all four limbs. Thyroid function was normal, and there were no clinical or radiological features suggestive of interstitial lung disease or other manifestations of anti‐synthetase syndrome. Muscle biopsy was not performed.

Based on these findings, a probable pembrolizumab‐induced polymyositis was diagnosed. Alternative etiologies, including immune‐related hypothyroid myopathy and paraneoplastic syndromes, were considered but deemed unlikely given the clinical course and laboratory results.

Treatment included intravenous methylprednisolone (40 mg/day) combined with intravenous immunoglobulins (0.4 g/kg). Dysphonia and dysphagia necessitated ENT consultation and nasogastric tube placement for enteral feeding.

A targeted cardiac evaluation for myocarditis (e.g., troponin testing or dedicated cardiology assessment) was not systematically performed. Although routine cardiac assessment may have been carried out during clinical management, myocarditis was not specifically investigated, representing a limitation of this report.

**FIGURE 1:**
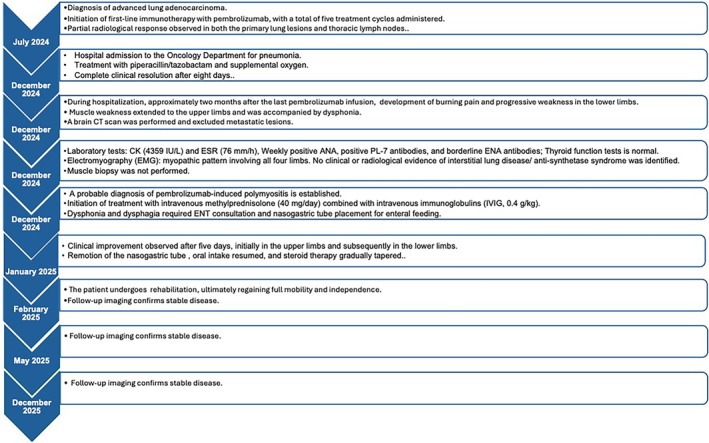
Timeline of diagnosis, treatment, and clinical outcomes of the presented patient. Case Report 1.

Clinical improvement was observed after 5 days, beginning in the upper limbs and subsequently involving the lower limbs. The nasogastric tube was removed, oral intake resumed, and steroid therapy was gradually tapered. The patient underwent rehabilitation, ultimately regaining full mobility and independence. She then continued clinical and radiological follow‐up, with stable disease at the last CT‐scan (December 2025) (Figure 1).

## 4 Case Report 2

A 78‐year‐old woman with a prior history of surgically treated breast ductal carcinoma (on letrozole) and basal cell carcinoma was diagnosed in May 2022 with high‐grade endometrial serous carcinoma. She underwent hystero‐oophorectomy and pelvic‐lumbar‐aortic lymphadenectomy, followed by adjuvant chemotherapy with carboplatin (AUC5) and paclitaxel, and radiation therapy (35 Gy in 5 fractions) to lumbar‐aortic lymphadenopathies.

In October 2022, she experienced abdominal lymph node recurrence and started first‐line therapy with pembrolizumab plus lenvatinib at the Department of Oncology, Sant'Anna Hospital, San Fermo della Battaglia, Italy.

From the second cycle, she developed grade 3 transaminitis (AST 131 IU/L, ALT 177 IU/L), mild CK elevation (343 IU/L), weakly positive ANA, negative ENA, and negative polymyositis panel. Clinically, she developed limb‐girdle weakness, limiting her ability to walk short distances independently.

Rheumatological evaluation suggested a probable immune‐related limb‐girdle myopathy. EMG revealed motor‐sensory polyneuropathy affecting both upper and lower limbs. Muscle biopsy was not performed. Alternative etiologies, including treatment‐related neuropathy and paraneoplastic phenomena, were considered but excluded based on the clinical context.

A targeted cardiac evaluation for myocarditis (e.g., troponin testing or cardiology assessment) was not performed in this case.

The patient was started on prednisone 25 mg daily; dose escalation to weight‐based recommendations was not tolerated due to hot flashes and fatigue. Due to her advanced age, comorbidities, and patient preference, IVIG and other steroid‐sparing immunosuppressive agents were not pursued, as the risk–benefit ratio was considered unfavorable.

**FIGURE 2:**
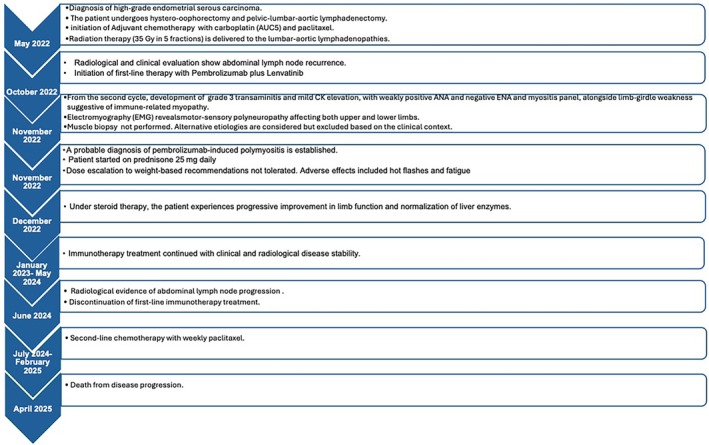
Timeline of diagnosis, treatment, and clinical outcomes of the presented patient. Case Report 2.

Under steroid therapy, she experienced progressive improvement in limb function and normalization of liver enzymes. Immunotherapy was continued until June 2024, when imaging revealed abdominal lymph node progression. The patient subsequently received weekly paclitaxel until February 2025 and died from progressive disease in April 2025 (Figure 2).

## 5 Discussion

Pembrolizumab‐induced myositis is a rare but clinically significant irAE associated with immune checkpoint inhibitor therapy. Although its overall incidence remains below 1% of all irAEs, literature reports indicate that most affected patients are older males, often with lung, urothelial, or skin cancers, presenting predominantly with proximal muscle weakness, elevated creatine kinase (CK) levels, and myopathic findings on EMG or imaging [2, 13, 14]. Severe presentations are frequently associated with bulbar involvement, respiratory muscle compromise, or concomitant myocarditis, which substantially increase morbidity and mortality.

In our series, the first patient, a 74‐year‐old woman with advanced lung adenocarcinoma, developed grade 4 myositis affecting both limb and phonatory muscles, raising concern for potential respiratory failure. The second patient, a 78‐year‐old woman with advanced endometrial carcinoma, presented with grade 3 limb‐girdle weakness. These cases highlight the most significant clinical challenges in managing pembrolizumab‐induced myositis: early recognition, accurate grading of severity, and timely intervention to prevent life‐threatening complications. Both cases also expand the known clinical spectrum, demonstrating that severe myositis can occur in female patients and in malignancies beyond the typical lung cancer demographic.

Pathophysiologically, pembrolizumab‐induced myositis is thought to result from multiple overlapping mechanisms. Pre‐existing autoimmune predisposition or autoantibodies may trigger exaggerated immune responses, while antigen spreading from tumor cell death can propagate cytotoxic T‐cell activation [15, 16]. Dysregulation between effector and regulatory T‐cells, and molecular mimicry between tumor and skeletal muscle antigens has been observed in biopsy specimens demonstrating infiltration by CD4+, CD8+ T‐cells, and macrophages, leading to multifocal necrosis [17, 18]. Both our cases lacked detectable myositis‐specific or associated autoantibodies, consistent with previous reports suggesting that de novo immune‐mediated myositis can occur independently of pre‐existing autoimmunity [3].

Clinically, most literature cases report progressive proximal weakness, myalgia, and fatigue, often with CK elevations proportional to disease severity. Ocular or bulbar involvement is observed in approximately 25% of cases and correlates with increased risk of concomitant myocarditis [2, 19].

In our first patient, the rapid progression to phonatory muscle involvement necessitated combined therapy with intravenous corticosteroids and IVIG, resulting in complete recovery. The second patient responded favorably to oral prednisone, demonstrating that therapeutic intensity can be tailored to symptom severity and patient tolerance. Both patients tolerated treatment without significant adverse events beyond expected corticosteroid‐related effects. Differential diagnoses, such as antibiotic‐induced myopathy, were carefully considered but deemed unlikely based on literature evidence and clinical course [20, 21].

Comparison with previously published cases emphasizes the variability of pembrolizumab‐induced myositis (Table 1). Among reported pembrolizumab‐specific cases identified from 2020 to 2025, most involved male patients, primarily with lung cancer, and commonly presented with four‐limb weakness and CK elevation. Bulbar or respiratory involvement, although less frequent, was associated with poorer outcomes and required more aggressive management. Our first case represents the only reported patient with severe phonatory involvement successfully treated with combined IV corticosteroids and IVIG, while the second case illustrates a favorable outcome in a patient with gynecologic malignancy, highlighting unique aspects not previously described in the literature. These findings reinforce that pembrolizumab‐induced myositis should be suspected across a broader demographic, including female patients and non‐lung tumors.

Diagnosis remains challenging, relying on integration of clinical, laboratory, and imaging findings. CK elevation, EMG with myopathic changes, and MRI or FDG‐PET/CT evidence of muscle inflammation are the primary diagnostic tools, while muscle biopsy remains the gold standard for definitive diagnosis but is reserved for atypical or refractory cases [10]. Early recognition and prompt initiation of immunosuppressive therapy are critical, as delayed intervention may result in irreversible muscle damage or fatal outcomes.

Management strategies should be individualized according to disease severity. Mild cases may respond to oral corticosteroids, whereas severe presentations with bulbar or respiratory involvement require high‐dose intravenous corticosteroids and, in selected cases, adjunctive IVIG or plasma exchange. Refractory cases may benefit from other immunosuppressants, including rituximab or IL‐6 inhibitors [22, 23]. Our cases demonstrate that early, tailored intervention within a multidisciplinary framework significantly improves clinical outcomes.

In conclusion, pembrolizumab‐induced myositis is a rare but potentially life‐threatening irAE. Our cases highlight several key clinical lessons: the importance of early recognition and grading of symptom severity, the integration of clinical, laboratory, and imaging data to distinguish myositis from tumor‐ or infection‐related weakness, and the utility of individualized immunosuppressive therapy. They also emphasize the heterogeneity of this toxicity, demonstrating that severe myositis can occur in female patients and in non‐lung malignancies. Early intervention can result in full recovery, even in grade 4 presentations, and a multidisciplinary approach is essential to optimize outcomes. Clinicians should maintain a high index of suspicion for ICI‐related myositis across diverse populations and carefully weigh risks and benefits when considering continuation of immunotherapy.

## 6 Conclusions

Myositis is an infrequent occurrence, but with a high lethality rate, increasingly described in patients undergoing immunotherapy treatment. Although today the information about its management is more precise and clearer, the diagnosis remains insidious as well as the possible treatments in case of refractoriness to steroid therapy. Further research will be necessary to help the clinician even more to orient himself in the correct management.

## Author Contributions

F.R. conceived the study. F.R. and N.L.A. performed the literature search and wrote the article. F.C., E.B., M.G., and P.P. critically revised the manuscript. All authors approved the final version of the manuscript.

## Funding

The authors have nothing to report.

## Consent

Written informed consent was obtained from all participants for participation in the study and for publication of the case details and any accompanying images.

## Conflicts of Interest

The authors declare no conflicts of interest.

## Data Availability

Data sharing not applicable to this article as no datasets were generated or analysed during the current study.

## References

[1] M. Reck , D. Rodríguez‐Abreu , A. G. Robinson , et al., “Pembrolizumab Versus Chemotherapy for PD‐L1‐Positive Non‐Small‐Cell Lung Cancer,” New England Journal of Medicine 375 (2016): 1823–1833. 27718847 10.1056/NEJMoa1606774

[2] Y. Allenbach , C. Anquetil , A. Manouchehri , et al., “Immune Checkpoint Inhibitor‐Induced Myositis, the Earliest and Most Lethal Complication Among Rheumatic and Musculoskeletal Toxicities,” Autoimmunity Reviews 19, no. 8 (2020): 102586, 10.1016/j.autrev.2020.102586. 32535094

[3] A. G. Solimando , L. Crudele , P. Leone , et al., “Immune Checkpoint Inhibitor‐Related Myositis: From Biology to Bedside,” International Journal of Molecular Sciences 21, no. 9 (2020): 3054, 10.3390/ijms21093054. 32357515 PMC7246673

[4] J. Claus , A. Van Den Bergh , S. Verbeek , E. Wauters , and K. Nackaerts , “Pembrolizumab‐induced necrotizing myositis in a patient with metastatic non‐small‐cell lung cancer: a case report,” Lung Cancer Management 8, no. 2 (2019), 10.2217/lmt-2018-0017. PMC6802710 31645893

[5] T. Yamano , M. Hamakawa , Y. Akaike , and T. Ishida , “A Case Report of Immune Checkpoint Inhibitor–Related Myositis and Cholangitis Induced by Pembrolizumab,” Clinical Case Reports 12, no. 7 (2024): e9153. 38962456 10.1002/ccr3.9153 PMC11220503

[6] B. Soman , M. C. Dias , S. A. J. Rizvi , and A. Kardos , “Myasthenia Gravis, Myositis and Myocarditis: A Fatal Triad of Immune‐Related Adverse Effect of Immune Checkpoint Inhibitor Treatment,” BMJ Case Reports 15, no. 12 (2022): e251966. 10.1136/bcr-2022-251966 PMC9743272 36593626

[7] S. Takahashi , K. Okabayashi , I. Soejima , A. Oniki , S. Ishihara , and H. Tomimitsu , “Pembrolizumab‐Induced Myopathy With Anti‐Striated Muscle Antibodies Successfully Treated by Plasma Exchange,” Internal Medicine 62, no. 23 (2023): 3525–3530. 38044116 10.2169/internalmedicine.1222-22 PMC10749821

[8] C. L. Haddox , N. Shenoy , K. K. Shah , et al., “Pembrolizumab Induced Bulbar Myopathy and Respiratory Failure With Necrotizing Myositis of the Diaphragm,” Annals of Oncology 28, no. 3 (2017): 673–675, 10.1093/annonc/mdw655. 27993808 PMC5391710

[9] S. Kobak , “Pembrolizumab‐Induced Seronegative Arthritis and Fasciitis in a Patient With Lung Adenocarcinoma,” Current Drug Safety 14, no. 3 (2019): 225–229. 31132977 10.2174/1574886314666190528121039 PMC6864613

[10] L. Peverelli , A. De Rosa , E. Domina , et al., “Severe inflammatory myopathy in a pulmonary carcinoma patient treated with Pembrolizumab: An alert for myologists,” Journal of Neuromuscular Diseases 7, no. 4 (2020): 511–514. https://doi.org.10.3233/JND‐200504. 32623405 10.3233/JND-200504

[11] H. Kamo , T. Hatano , K. Kanai , et al., “Pembrolizumab‐Related Systemic Myositis Involving Ocular and Hindneck Muscles Resembling Myasthenic Gravis: A Case Report,” BMC Neurology 19 (2019): 184. 31382909 10.1186/s12883-019-1416-1 PMC6681482

[12] J. Aldrich , X. Pundole , S. Tummala , et al., “Inflammatory Myositis in Cancer Patients Receiving Immune Checkpoint Inhibitors,” Arthritis & Rhematology 73, no. 5 (2021): 866–874, 10.1002/art.41604. 33258544

[13] N. Hamada , A. Maeda , K. Takase‐Minegishi , et al., “Incidence and Distinct Features of Immune Checkpoint Inhibitor‐Related Myositis From Idiopathic Inflammatory Myositis: A Single‐Center Experience With Systematic Literature Review and Meta‐Analysis,” Frontiers in Immunology 6, no. 12 (2021): 803410, 10.3389/fimmu.2021.803410. PMC8686164 34938300

[14] A. Ribas and J. D. Wolchok , “Cancer Immunotherapy Using Checkpoint Blockade,” Science 359 (2018): 1350–1355. 29567705 10.1126/science.aar4060 PMC7391259

[15] J. Wang , Y. Ma , H. Lin , J. Wang , and B. Cao , “Predictive Biomarkers for Immune‐Related Adverse Events in Cancer Patients Treated With Immune‐Checkpoint Inhibitors,” BMC Immunology 25, no. 1 (2024): 8, 10.1186/s12865-024-00599-y. 38267897 PMC10809515

[16] D. B. Johnson , J. M. Balko , M. L. Compton , et al., “Fulminant Myocarditis With Combination Immune Checkpoint Blockade,” New England Journal of Medicine 375, no. 18 (2016): 1749–1755, 10.1056/NEJMoa1609214. 27806233 PMC5247797

[17] A. Matas‐García , J. C. Milisenda , A. Selva‐O'Callaghan , et al., “Emerging PD‐1 and PD‐1L Inhibitors‐Associated Myopathy With a Characteristic Histopathological Pattern,” Autoimmunity Reviews 19, no. 2 (2020): 102455, 10.1016/j.autrev.2019.102455. 31838162

[18] K. Sekiguchi , R. Hashimoto , Y. Noda , et al., “Diaphragm Involvement in Immune Checkpoint Inhibitor‐Related Myositis,” Muscle & Nerve 60, no. 4 (2019): E23–E25, 10.1002/mus.26640. 31323130

[19] L. Janssen , N. A. E. Allard , C. G. J. Saris , J. Keijer , M. T. E. Hopman , and S. Timmers , “Muscle Toxicity of Drugs: When Drugs Turn Physiology Into Pathophysiology,” Physiological Reviews 100, no. 2 (2020): 633–672. 31751166 10.1152/physrev.00002.2019

[20] S. Miernik , A. Matusiewicz , and M. Olesińska , “Drug‐Induced Myopathies: A Comprehensive Review and Update,” Biomedicine 12, no. 5 (2024): 987, 10.3390/biomedicines12050987. PMC11117896 38790948

[21] M. C. Dalakas , I. Illa , J. M. Dambrosia , et al., “A Controlled Trial of Intravenous Immunoglobulin in the Treatment of Chronic Inflammatory Demyelinating Polyneuropathy,” New England Journal of Medicine 329 (1993): 1145–1151.

[22] J. Leipe and X. Mariette , “Management of Refractory Autoimmune Myositis,” Rheumatology (Oxford, England) 58 (2019): v21–v28. 10.1093/rheumatology/kez360 PMC6900914 31816078

[23] O. M. Uryas’yev , O. Y. Lazareva , A. V. Shakhanov , et al., “Use of Intravenous Immunoglobulin in Treatment for Refractory Dermatomyositis: A Case Report//I.P,” Pavlov Russian Medical Biological Herald 33, no. 1 (2025): 105–115, 10.17816/PAVLOVJ456412.

